# Hypoglycemic Effect of Exopolysaccharide from *Lactiplantibacillus plantarum* JLAU103 on Streptozotocin and High-Fat Diet-Induced Type 2 Diabetic Mice

**DOI:** 10.3390/foods11223571

**Published:** 2022-11-09

**Authors:** Yuan Qi, Danyang Wang, Li Fang, Xiaoting Liu, Chunlei Liu, Fanrui Zhao, Dan Wu, Xiyan Wang, Ji Wang, Weihong Min

**Affiliations:** College of Food Science and Engineering, Jilin Agricultural University, Changchun 130118, China

**Keywords:** *Lactiplantibacillus plantarum*, exopolysaccharide, type 2 diabetic, hypoglycemic, hypolipidemic, neuroprotective

## Abstract

Two doses (300 mg/kg bw and 600 mg/kg bw) of the *Lactiplantibacillus plantarum* JLAU103 exopolysaccharide (EPS103) were orally administered to a type 2 diabetic (T2DM) mouse model induced by streptozotocin and a high-fat diet. The hypoglycemic, hypolipidemic and neuroprotective effects of EPS103 on T2DM mice were evaluated. The results indicated that administration of EPS103 could alleviate insulin resistance, reduce the levels of fasting blood glucose, glycosylated hemoglobin A1c, leptin and fasting serum insulin, improve glucose tolerance, protect pancreas and liver, and modulate blood lipid disorders. EPS103 promoted hepatic glycogen synthesis by upregulating the phosphorylation of GSK3β. Meanwhile, it upregulated the phosphorylation of IRS-1, PI3K and Akt, as well as the expression of IRS-2 and GLUT4, and downregulated the expression of PEPCK, G6Pase and PGC-1α, indicating that EPS103 promotes the uptake and transport of glucose and inhibits gluconeogenesis, which might be related to the activation of the IRS-1/PI3K/Akt pathway. Additionally, EPS103 can protect against brain nerve damage through improving oxidative stress injury, restoring the expression of IRS-2, alleviating neuronal apoptosis and inhibiting inflammation in the hippocampus of T2DM mice. Taken together, our results demonstrated that EPS103 may be a potential therapeutic agent for the treatment of T2DM.

## 1. Introduction

Hyperglycemia and insulin resistance are the hallmarks of type 2 diabetes mellitus (T2DM). The complications of T2DM include cardiovascular disease, renal failure, retinopathy, cognitive function, and mental health disorders. The advantage of commercially available hypoglycemic drugs, such as biguanides, sulfonylureas, and α-glycosidase inhibitors, is that there is an obvious effect of lowering blood sugar. However, the disadvantage is that oral administration of such drugs often causes side effects such as gastrointestinal problems, decreased appetite, dizziness, and vomiting [1]. Considering the long-term course of T2DM treatment, these issues should be carefully considered. Therefore, the search for alternatives to traditional hypoglycemic agents, such as natural products and functional foods with milder side effects, is urgently needed.

In the presence of glucose, streptozotocin (STZ), a glucosamine nitrosourea, enters cells exclusively through the glucose transporter type 2 (GLUT2). STZ has a selective damaging effect on pancreatic islet β cells, which results in defective insulin signaling, IR, and other phenomena in mice. It has been reported that STZ is neurotoxic and can also lead to neuronal loss and thinning of the parietal cortex in the mouse brain. Thus, STZ is widely used to induce T2DM in mice. STZ-induced IR not only affects glucose metabolism in the hippocampus and cortex, but also leads to activation of microglia, leading to inflammatory responses, oxidative stress, synaptic dysfunction, and other Alzheimer’s disease-like pathological features. All mammalian cells require glucose as a metabolic substrate. In the brain, glucose is the primary source of energy, as it cannot be synthesized or stored in sufficient quantities to maintain cellular function. The steady supply of glucose to the brain is determined by the levels of hexose sugars circulating in the blood. Therefore, the availability of blood glucose levels in normal human circulation must be tightly controlled to avoid large changes. The liver plays a major role in regulating glucose metabolism, including the balance between glucose uptake and storage through glycogenesis, and glucose release through glycogenolysis and gluconeogenesis [2]. Most of the mechanisms required to regulate blood glucose levels are peripheral, but the central nervous system (CNS) is known to play an important role in controlling circulating blood glucose. A key role played by the autonomic nervous system (ANS) in regulating blood glucose homeostasis in the liver, pancreas, and adrenal gland is the regulation of many physiological processes [3]. It is clear from this that glucose is used in the brain in an insulin-independent manner, contrary to observations in the periphery. Thus, the CNS is an insulin-insensitive organ. It is important to recognize, however, that the brain contains a large number of insulin receptors [4]. Additionally, in the CNS, insulin has many functions, such as being involved in learning, memory, neuronal growth and maturation [5]. Notably that insulin plays an important role in the hypothalamus and inhibits the release of glucose from the liver [6].

Exopolysaccharide (EPS) is a carbohydrate compound that is produced and secreted during mucilage secretion when microorganisms grow. In recent years, EPS derived from Lactobacillus have attracted the attention of researchers due to their diverse biological activities, safety, and short production cycles [7]. In particular, related studies have reported that EPS from *Lactiplantibacillus plantarum* has potential hypoglycemic activity. Dilna et al. demonstrated that the EPS from *L. plantarum* RJF4 displayed 40% inhibition of α-amylase and 42.24% decrease in cholesterol at a concentration of 800 μg/mL via an in vitro anti-diabetic evaluation assay [8]. Moreover, EPS from *L. plantarum* H31 promoted glucose uptake and upregulated the expression of glycometabolism-related proteins, including GLUT4, Akt2, and AMPK in IR HepG2 cells [9]. Taken together, these studies have focused on the inhibitory effect of EPS on α-amylase in vitro. As far as we know, no studies have been conducted using T2DM mice to evaluate EPS103 from L. plantarum on glycemic metabolism. Therefore, the present study aimed to explore the role of EPS103 in insulin resistance, glucose metabolism, and brain hippocampal damage in T2DM mice induced by STZ supplementation with a high-fat diet (HFD).

## 2. Materials and Methods

### 2.1. Materials and Reagents

Jilin Agricultural University’s Fermentation Engineering Laboratory provided EPS103 from L plantarum JLAU103 (Changchun, China). Primary antibodies against insulin receptor substrate-1 (IRS-1), phosphated IRS-1 (p-IRS-1), glycogen synthase kinase-3β (GSK-3β), phosphated GSK-3β (p-GSK-3β), phosphoenolpyruvate carboxykinase (PEPCK), glucose-6-phosphatase (G6Pase), peroxisome proliferator-activated receptor-gamma co-activator-1alpha (PGC-1α), glucose transporter type 4 (GLUT4), insulin receptor substrate-2 (IRS-2), nuclear factor-κB (NF-κB) and β-actin were purchased from Abcam (Cambridge, UK). Primary antibodies against phosphoinositide 3-kinase (PI3K), phosphated PI3K (p-PI3K), protein kinase B (Akt), phosphated Akt (p-Akt), c-Fos and peroxisome proliferator-activated receptor gamma coactivator-1 alpha (PGC-1α) were purchased from Cell Signaling Technology Inc. (Danvers, MA, USA). A set of anti-G6Pase antibodies was acquired from Boasen Biotechnology Co., Ltd. (Beijing, China). AB Cloning Technologies (Wuhan, China) provided the horseradish peroxidase (HRP)-conjugated secondary antibodies. The following assay kits were purchased from Jiancheng Biological Engineering Institution (Nanjing, China): superoxide dismutase (SOD), phospholipid hydroperoxide glutathione peroxidase (GSH-Px), catalase (CAT), malondialdehyde (MDA), triglycerides (TG), total cholesterol (TC), LDL cholesterol, HDL cholesterol, and BCA protein assay kits. R&D Systems (St. Louis, MO, USA) provided ELISA kits for glycosylated hemoglobin A1C (HbA1c), leptin (LEP) and fasting serum insulin (FINS). In order to obtain HFD, we purchased it from Beijing Darwincell Biotechnology Co., LTD (Catalog No.: H10052; Beijing, China). The rest of the analytical-grade substances were commercially available.

### 2.2. Animals and Experiment Design

The four weeks old C57BL/6J mice (male, 20 ± 2 g) used in this study were from Liaoning Changsheng Biotechnology Co., Ltd. (Certificate No.: SXCK (Liao) 2020-0001, Shenyang, China). This study was approved by the committee for the protection of animal care at the laboratory animal center of Jilin Agricultural University (Animal use license No.: SYXK (Ji) 2018-0023). The mice were kept in the animal room (12/12 h light dark cycle, free drinking and eating), with 10 mice in each cage. The feeding environment temperature was 23 ± 3 °C and the relative humidity was 40–70%. After one week acclimatization, the mice were randomly divided into five groups with 10 mice in each group: a normal control group (NC), a model control group (MC), a positive control group (PC), a low-dose treatment group (LD) and a high-dose treatment group (HD).

After one week acclimatization, in the second week, except for the NC group, the other groups of mice were given a HFD for 5 weeks. On the first day of the seventh week, mice in the NC group were injected intraperitoneally with an appropriate amount of sodium citrate buffer (pH 4.5) based on their body weight (BW) for three consecutive days. Additionally, the mice in the other five groups were injected intraperitoneally with 50 mM STZ at a dose of 50 mg/kg bw for three consecutive days. After three days of the last dose, measurement of fasting blood glucose (FBG) levels in mice with a blood glucose meter. (Accu-Chek Go, Roche Applied Science, Indianapolis, IN, USA) in order to determine the successful establishment of a T2DM model. The mice with FBG levels above 11.1 mmol/L were considered as successfully induced T2DM mice. Subsequently, mice were given intraperitoneal treatment every day for 4 weeks. Normal saline was given to NC and MC groups, metformin was given to PC [10], and EPS103 was given to LD and HD groups, respectively. Blood from the orbital sinus was collected at the end of the experiment and immediately centrifuged for 5 min at 4 °C at 3000 g. The serum was separated and stored at −80 °C until analyses. The pancreas, liver, and hippocampus were then collected after mice were sacrificed by cervical dislocation. Hematoxylin and eosin (H&E) staining was used to examine the pathological histology of the pancreas and liver. The rest tissues were stored at −80 °C until analyses. Organ indexes were calculated as follows [10]:

$$\mathrm{Organ}\;\mathrm{index}=\frac{\mathrm{average}\;\mathrm{weight}\;\mathrm{of}\;\mathrm{organ}\;\left(g\right)}{\mathrm{body}\;\mathrm{weight}\;\left(g\right)}\times 100\%$$

### 2.3. The Oral Glucose Tolerance Test (OGTT)

The oral glucose tolerance test was performed the beginning of the first week and at the end of the fourth week. All mice were fasted for 12 h, and then blood glucose levels at 0, 30, 60, and 120 min were analyzed by tail blood cutting [11]. Additionally, then calculate the area under the glucose curve (AUC-OGTT).

### 2.4. Blood Biochemical Measurement

The contents of HbA1c, LEP and insulin in serum were calculated by using commercial ELISA kits. The lipids including TG, TC, LDL-C, and HDL-C were measured by using commercial kits. All analyses were performed in accordance with the manuals provided by the manufacturers. The homeostasis model assessment of IR (HOMA-IR) index was calculated as follows [11]:

$$\mathrm{HOMA}-\mathrm{IR}\;\mathrm{index}\;=\frac{\mathrm{FBG}\left(\mathrm{mmol}/L\right)\times \;\mathrm{FIN}\left(\mathrm{mIU}/L\right)}{22.5}$$

### 2.5. Determination of Hepatic Glycogen Synthesis

Liver tissues were fixed in 4% paraformaldehyde, embedded in paraffin, and sectioned (4 µm). Then, the sections were used for PAS staining according to the method described by Wei et al. [12] The images were captured using an Olympus microscope (Olympus, Japan), the positive area stained relative to the plaque area was calculated using Image Pro Plus 6.0 software (Media Cybernetics). Additionally, the content of hepatic glycogen was determined by using the glycogen assay kit follow by the manufacturer’s protocols.

### 2.6. Determination of Antioxidant Activity in Hippocampus

Using a tissue disruptor, hippocampal tissues were homogenized with normal saline. In accordance with the manufacturer’s instructions, kits were used to determine the activities of SOD, GSH-Px, and CAT, as well as the content of MDA.

### 2.7. Western Blot Assay

Protein extraction kits are used to extract total cellular protein from tissues (Beyotime, Shanghai, China). Protein concentration was identified via a BCA test kit. Additionally, this was followed by 2× denaturing buffer treatment for 10 min. Following 10% and 12% sodium dodecyl-sulfate polyacrylamide gel electrophoresis, afterward moved onto the PVDF membrane (Dingguo Changsheng Biological Technology Company, Beijing, China), sealed with 5% skimmed milk powder for 1 h, the membrane was incubated with primary antibody in a shaker flask at 4 °C overnight and then incubated with secondary antibody at room temperature for 1 h. The cleaning of the films was performed with Tris buffered saline Tween (TBST); afterward, the films were cultivated in HRP-conjugated secondary antibodies for 60 min. Finally, the electrochemiluminescence exposure solution was evaluated using laboratory imaging software. *β*-actin was used as an internal reference.

### 2.8. Statistical Analysis

Every assay was completed three or five times, and we determined the group mean and standard deviation, and also performed one-way ANOVA. The resulting data are presented as graphs and expressed as the mean ± standard deviation (SD). *p* < 0.05 was considered statistically significant, and graphs were generated using Origin Pro 8.5.

## 3. Results and Discussion

### 3.1. The Effect of EPS103 on FBG, Lipids and the OGTT

FBG levels were measured for 7 weeks. As shown in Table 1, the average FBG level in the NC group was stable at 5 mmol/L throughout the feeding period. The average FBG level in the MC group remained at 12 mmol/L during the feeding period, which was significantly higher than that of the NC group (*p* < 0.05), indicating that STZ and an HFD successfully induced and simulated hyperglycemia in mice. In all treatment groups, FBG levels decreased starting at the eleventh week, with FBG levels decreasing by 36.01%, 36.09%, and 50.04%, respectively, in the PC, LD, and HD groups, in comparison to the MC group (*p* < 0.05). These data suggest that EPS103 had a positive effect on FBG levels in diabetic mice, and high-dose treatment resulted in better hypoglycemic effects.

It was also studied whether EPS103 would cause hypolipidemia in mice following oral administration. A significant difference was found in serum TG, TC, and LDL-C levels between MC and NC mice in Table 2 (*p* < 0.05). After T2DM mice were treated with EPS103 and metformin, the levels of TC, TG, and LDL-C significantly decreased in each group (*p* < 0.05). Compared to MC mice, TG concentrations in high-dose and low-dose groups of EPS103 were significantly decreased by 35.86% and 44.14% (*p* < 0.05), TC concentrations decreased by 10.51% and 11.56% (*p* < 0.05), and LDL-C concentrations decreased by 30.15% and 31.66% (*p* < 0.05), respectively. Furthermore, serum HDL-C levels were significantly lower in the MC group than in the NC group (*p* < 0.05). Serum HDL-C concentrations increased significantly (*p* < 0.05) following administration of EPS103. EPS103 significantly increased HDL-C concentrations in both the high-dose and low-dose groups when compared with MC mice (*p* < 0.05). Our results suggest that EPS103 may contribute to metabolic homeostasis in relation to lipids and blood glucose.

**Figure 1:**
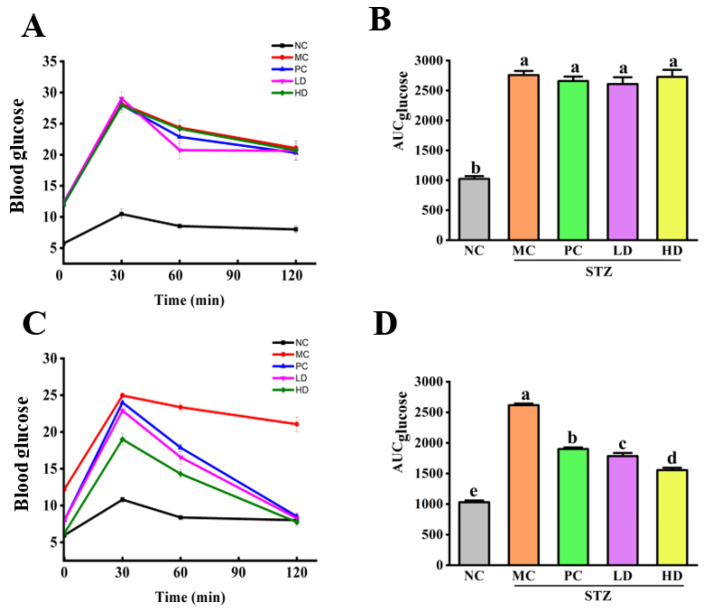
Effect of EPS103 on glucose tolerance in the T2DM mice induced by STZ and HFD. (**A**) OGTT and (**B**) AUC on the first day of the first week of intragastric administration. (**C**) OGTT and (**D**) AUC on the sixth day of the fourth week of intragastric administration. Data are presented as the mean ± SD (n = 5). Different letters indicate statistically significant differences between two groups (*p* < 0.05).

Figure 1 shows the changes in the blood glucose levels during the OGTT. After intraperitoneal injection of STZ for 3 days, the blood glucose level (Figure 1A) and area under curve (AUC) of the OGTT (Figure 1B) of the MC and treatment groups (PC, LD, and HD groups) were similar and were significantly higher than those of the NC group (*p* <0.05). The blood glucose levels in all groups increased to a maximum 30 min, and then decreased over the following 90 min. The blood glucose levels of the mice in the NC group returned to their original levels 120 min after the oral glucose challenge. However, the blood glucose levels in the MC and treatment groups (PC, LD, and HD) were all higher than the original levels 120 min after the oral glucose challenge. The tests were remarkably lower in the treatment groups than those in the MC group at every time point after 4 weeks of treatment (Figure 1C) and were significantly lower than those of the first day of treatment (*p* < 0.05). Among the EPS103 treatment groups, the mice treated with higher doses had more potent glucose tolerance than mice treated with lower doses. These results indicated that EPS103 intervention effectively lowered blood glucose levels in T2DM mice by improving glucose tolerance. Consistent with this, Sun et al. [13] and Wang et al. [11] found that the EPS from *Cordyceps militaris* and *Lachnum* YM240, could reduce blood glucose concentration in STZ and HFD-induced T2DM mice, respectively, resulting in the relief of diabetes.

### 3.2. The Effect of EPS103 on HbA1c, LEP, FINS and HOMA-IR

**Figure 2:**
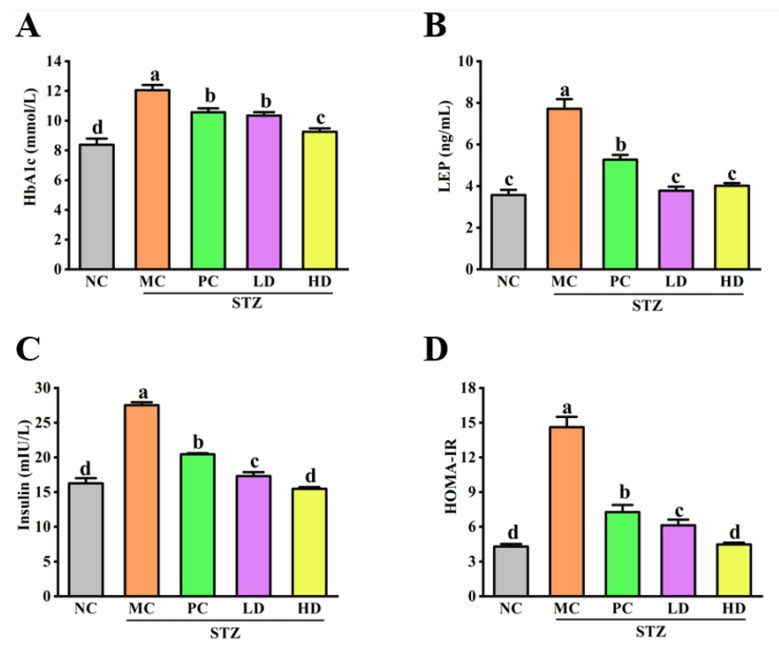
Effect of EPS103 on IR in the T2DM mice induced by STZ and HFD. (**A**) HbA1c. (**B**) LEP. (**C**) FINS. (**D**) HOMA-IR index. Data are presented as the mean ± SD (n = 5). Different letters indicate statistically significant differences between two groups (*p* < 0.05).

HbA1c, as an important indicator to detect long-term blood glucose, reflects the average blood glucose concentration in serum during 2-3 months, and the relationship between IR and blood glucose. As shown in Figure 2A, HbA1c levels in the MC group were significantly higher than those in the NC group (*p* < 0.05). After administration of EPS103, the HbA1c levels in the LD and HD groups were significantly decreased (*p* < 0.05), with the percentage fall rates of 14.2% and 23.2%, respectively, as compared to the MC group. LEP has been shown to reduce hyperglycemia in T1DM and has recently been shown to normalize fasting plasma glucose concentrations in polygenic obesity and T2DM. Overall, these findings suggest that LEP may be an effective therapeutic option for diabetes. According to Figure 2, the FINS level for the MC group was significantly higher than the FINS level for the NC group by 10 mIU/L (*p* < 0.05); however, after administration of EPS103, FINS levels were significantly decreased (*p* < 0.05), suggesting that administration of EPS103 was associated with increased insulin sensitivity, reducing serum FINS levels.

IR is a distinctive feature of T2DM and is defined as a reduced response of cells or tissues to physiological levels of insulin [14]. Advanced hyperinsulinemia is thought to be characteristic of diabetic mice, so the effect of EPS103 on FINS was investigated. As shown in Figure 2C, FINS level in MC group was significantly higher than in NC group by 10 mIU/L (*p* < 0.05); however, after administration of EPS103, FINS levels were significantly decreased (*p* < 0.05), which indicated that administration of EPS103 could enhance the sensitivity of the body to insulin, thereby reducing the FINS levels in serum. HOMA-IR, the gold standard for evaluating IR, is derived from FBG values together with FINS. The HOMA-IR index results are shown in Figure 2D; the HOMA-IR index of the MC group was 14.6 ± 0.9, which was significantly higher than that of the NC group (*p* < 0.05). Both EPS103 and metformin significantly decreased the HOMA-IR index compared to that of the MC group (*p* < 0.05). In particular, treatment with a high dose of EPS103 restored the HOMA-IR index to a level similar to that in NC mice. Taken together, these results indicate that EPS103 administration could effectively reverse the hyperglycemia induced by STZ and HFD by improving IR.

### 3.3. EPS103 Ameliorates Pancreatic and Hepatic Injury

**Figure 3:**
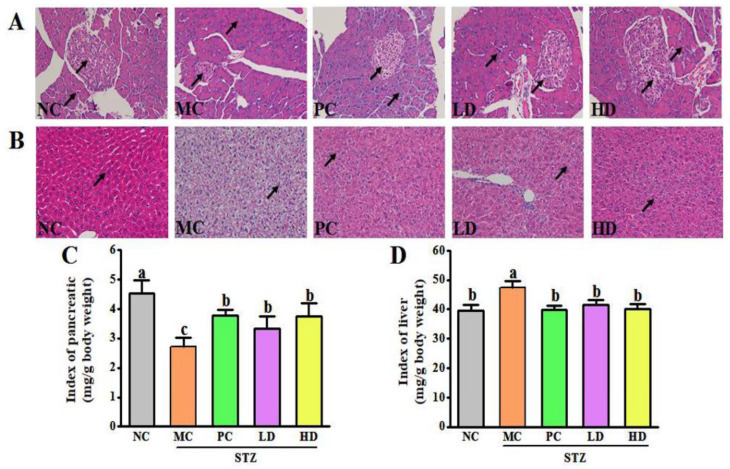
Effect of EPS103 on pancreas and liver injury in the T2DM mice induced by STZ and HFD. (**A**) H&E staining of pancreas. Scale bars = 100. (**B**) H&E staining of liver. Scale bars = 100 μm. (**C**) Pancreas index. (**D**) Liver index. Data are presented as the mean ± SD (n = 5). Different letters indicate statistically significant differences between two groups (*p* < 0.05).

To evaluate the protective effects of EPS103 on pancreatic and hepatic injury induced by STZ and HFD, H&E staining was performed and organ indices of the pancreas and liver were analyzed. The changes in pancreatic histology for the different groups are presented in Figure 3A. In the pancreatic tissue of the NC group, pancreatic cells were neatly arranged and evenly distributed, presenting round or oval islets that were dyed light pink. In the group receiving MC, there was evident degeneration of the pancreatic tissue. Specifically, the pancreatic tissue of the MC group showed blurred islet contours, irregular distribution of pancreatic cells, obvious gaps and vacuoles, and significant changes in pancreatic morphology and islet number. The pancreatic index of the MC group was significantly lower than that of the NC group (Figure 3C, *p* < 0.05). The severe pancreatic lesions may be due to increased blood glucose concentrations that lead to apoptosis of pancreatic cells, decreased insulin secretion, and enhanced cell secretion of glucagon. However, these pancreatic lesions were considerably diminished by the administration of metformin and EPS103 (LD and HD). The metformin and EPS103 treatment groups showed significantly greater improvements in the morphology of pancreatic cells, the number of islets, and the pancreatic index (*p* < 0.05) when compared to the MC group. The histology of the liver for the different groups is shown in Figure 3B. In the NC group, the morphology of hepatocytes was normal, the nucleus was found in the middle, the liver tissue structure was evident, the central vein was clearly visible, and there were no obvious pathological changes. There were relatively uniform sizes and morphologies of liver cells in the NC group, as well as neatly arranged and evenly distributed cells. However, most hepatocytes showed diffuse vesicular steatosis, and some hepatocytes were bulky and edematous, with cell changes, disordered cell arrangements, and local necrosis, with an infiltrating inflammatory response. Furthermore, the liver index of the MC group was significantly higher than that of the NC group (*p* < 0.05). This suggests that the increase in blood glucose concentration leads to liver damage, decreased liver function, and disorders of the synthesis of glycogen and other processes. However, these liver lesions were considerably diminished by the administration of metformin and EPS103 (LD and HD). Treatment groups (PC, LD, and HD) demonstrated significant improvement in the morphology of liver cells compared to the MC group, as well as a significant increase in the liver index. In addition to improved hepatocyte arrangement and nuclear structure, significant improvements were also noted in liver fat and degree of damage. However, the recovery effect of the EPS103 treatment group (LD and HD) at different concentrations was similar, with no significant difference. T2DM is a chronic metabolic disorder and the liver is an important metabolic organ that is closely associated with T2DM. Common hepatic complications of T2DM include liver cancer, as well as various types of fatty liver disease [15]. The formation of fatty liver in patients also further contributes to the development of IR [16,17]. The body can produce a variety of hormonal species to promote the elevation of blood glucose, but insulin secreted by pancreatic islet *β* cells is the only polypeptide hormone with hypoglycemic efficacy. It has been shown that glucotoxicity due to T2DM accelerates apoptosis of pancreatic islet *β* cells [18], and pancreatic health directly affects the body’s ability to regulate blood glucose. Our results showed that compared with NC mice, MC mice had significantly enlarged livers with significantly increased liver indices, which were significantly restored to levels close to healthy and significantly reduced tissue lesions after EPS103 gavage treatment. Intragastric administration of EPS103 significantly ameliorated pancreatic injury in T2DM mice, consistent with previous findings. Pan et al. (2014) showed that *Dendrobium officinale* polysaccharides reduced FBG levels and elevated body antioxidant capacity by alleviating pathological damage in the pancreatic islets of T2DM mice [19].

### 3.4. The Effect of EPS103 on Hepatic Glycogen Synthesis

**Figure 4:**
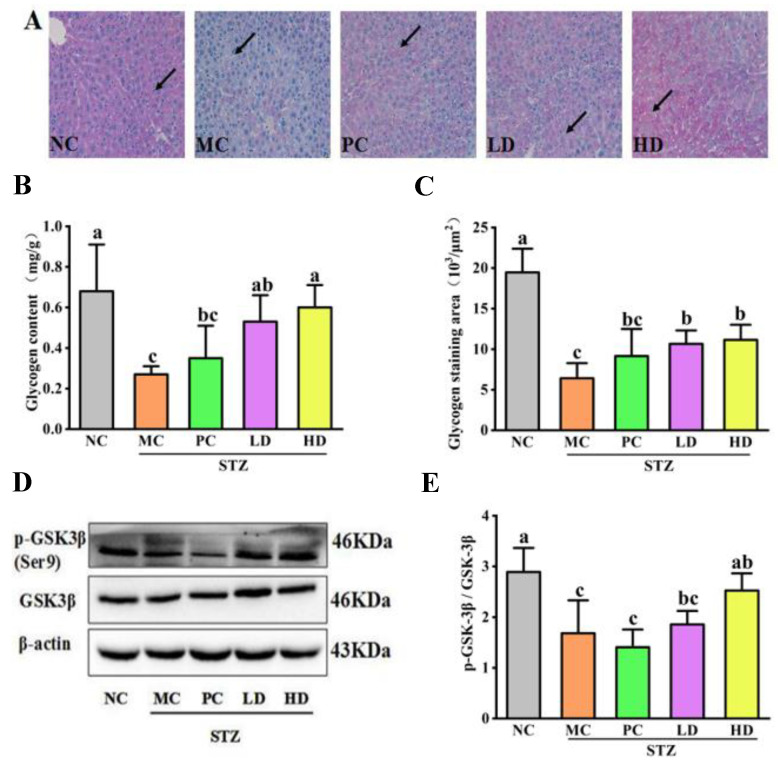
Effect of EPS103 on hepatic glycogen synthesis in the T2DM mice induced by STZ and HFD. (**A**) PAS staining of liver. Scale bars = 100 μm. (**B**) Glycogen content. (**C**) Glycogen staining area. (**D**) Bands of GSK-3*β* and *p*- GSK-3*β* (Ser9). (**E**) Protein expression levels of phosphorylated GSK-3*β*. *β*-actin was utilized as loading control. Data are presented as the mean ± SD (n = 3). Different letters indicate statistically significant differences between two groups (*p* < 0.05).

Hepatic glycogen is a substance formed by the polymerization of many glucose molecules, which are stored in the liver as glycogen and could be broken down into glucose for energy conversion when the body needs it [20]. A normal diet can continuously replenish liver glycogen in healthy individuals to reduce gluconeogenesis and also produce better preserved proteins in vivo. To measure glycogen synthesis in the liver of T2DM mice using PAS glycogen staining, we determined glycogen synthesis using PAS glycogen staining (Figure 4A). When liver cells were stained with PAS, they appeared pink in the NC group. Glycogen staining was not evident in mice from the MC group. EPS103 and metformin significantly increased liver glycogen staining in mice, particularly in those treated with HD. Additionally, we performed statistical analysis of the staining area of glycogen content in the liver of the mice in the MC group, and we found that the glycogen content and glycogen staining area in the liver were significantly lower than those in the NC group (*p* < 0.05, Figure 4B,C). This shows that a decline in hepatic glycogen anabolic metabolism is an important pathological mechanism of diabetes. Compared with the MC group, the glycogen content and glycogen staining area were significantly increased after treatment of EPS103 (*p* < 0.05). Consistently, the recovery effect was more pronounced in the HD group. This indicates that EPS103 can promote glycogen synthesis in the liver of T2DM mice. 

Furthermore, we explored the mechanism by which EPS103 affects glycogen synthesis. We investigated the expression of GSK-3*β* and its Ser9 phosphorylated proteins. GSK-3*β* is a key enzyme involved in and associated with glycogen synthesis. The literature indicates that IR inhibits GSK-3*β* phosphorylation level in the liver, which in turn leads to decreased levels of hepatic glycogen synthesis in mice with T2DM [21]. GSK-3*β* and the phosphorylation at Ser9 results in a decrease in the glycogen synthase (GS) phosphorylation level, increased GS activity, increased glycogen synthesis, and significantly reduced blood glucose concentration. In the MC group, Ser9 phosphorylation of GSK-3*β* decreased significantly (*p* < 0.05; Figure 4D,E). The mice treated with high-dose EPS103 had a significantly increased Ser9 phosphorylation level of GSK-3β compared with the MC group (*p* < 0.05). Based on the above results, we can infer that EPS103 can promote glycogen synthesis by upregulating the Ser9 phosphorylation of GSK-3*β* in the liver of T2DM mice, resulting in the effect of reducing blood glucose.

### 3.5. The Effect of EPS103 on Glucose Uptake

**Figure 5:**
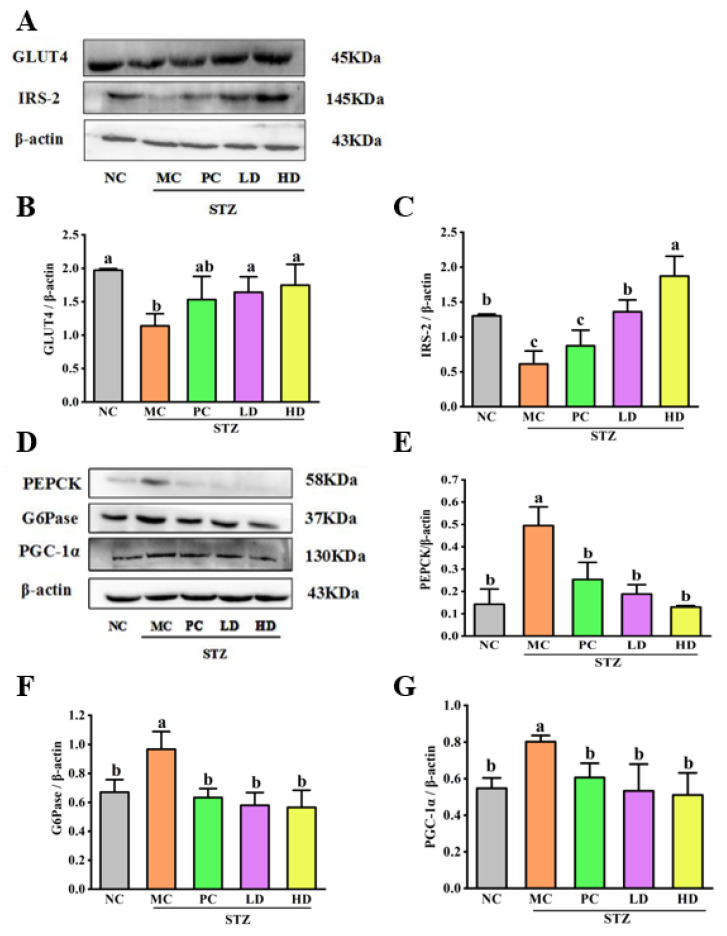

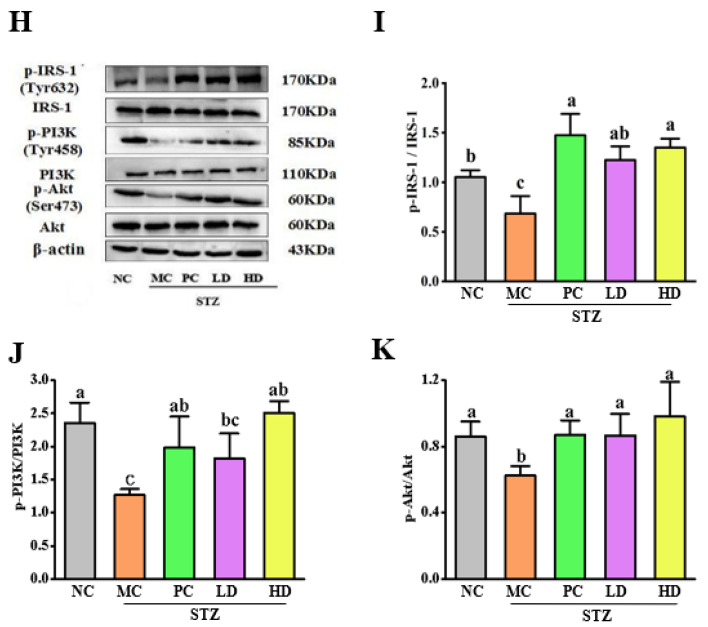
Effects of EPS103 on glucose uptake, gluconeogenesis and the IRS-1/PI3K/Akt pathway in the T2DM mice induced by STZ and HFD. (**A**) Bands of GLUT4 and IRS-2. Protein expression levels of (**B**) GLUT4 and (**C**) IRS-2. (**D**) Bands of PEPCK, G6Pase and PGC-1α. Protein expression levels of (**E**) PEPCK, (**F**) G6Pase and (**G**) PGC-1α. (**H**) Bands of IRS-1, p-IRS-1 (Tyr632), PI3K, p-PI3K (Tyr458), Akt and p-Akt (Ser473). Protein expression levels of phosphorylated (**I**) IRS-1, (**J**) PI3K and (**K**) Akt. *β*-actin was utilized as loading control. Data are presented as the mean ± SD (n = 3). Different letters indicate statistically significant differences between two groups (*p* < 0.05).

As the main functional molecule of the human body, glucose needs to be carried by transporters into cells. There are four glucose transporter proteins, among which GLUT4 is the main protein to assist glucose transport. GLUT4 can take up external glucose and transport it into the cell interior, reducing blood glucose levels. Thus, the loss of GLUT4 ultimately leads to transport dysfunction and abnormal glucose metabolism [22]. Insulin receptor substrates (IRS) are important signaling proteins at post-receptor levels in the insulin signaling pathway, and four insulin receptor substrates have been studied. IRS-2 is usually expressed in large quantities in the liver and pancreatic *β* cells. Defects in the expression of IRS-2 induces IR at the hepatic site. In this study, we used Western blot to detect levels of GLUT4 and IRS-2 in the liver of T2DM mice. According to Figure 5A,B, GLUT4 expression was significantly decreased in the MC group compared to the NC group (*p* < 0.05). As a result, we found that GLUT4 expression was significantly increased in mice treated with EPS103 as compared with those treated with MC (*p* < 0.05); recovery was better in the EPS103 treatment groups than in the PC treatment groups, but there were no significant differences between the two groups. In summary, the expression of GLUT4 in the EPS103 treatment groups significantly enhanced glucose uptake in T2DM mice. The levels of IRS-2 (0.6 ± 0.2) in the MC group were significantly suppressed (Figure 5A,C). However, significant hepatic IRS-2 recovery was achieved in mice treated with EPS103 compared to the MC group. It can be clearly seen that EPS103 processing is more efficient than in the PC group. The results showed that the regulation of GLUT4 and IRS-2 affected glucose homeostasis and insulin sensitivity in T2DM mice. EPS103 treatment groups were able to regulate blood glucose levels by restoring glucose transport and uptake levels in mouse liver tissue.

### 3.6. The Effect of EPS103 on Gluconeogenesis

Elevated gluconeogenesis levels in individuals with T2DM increase the amount of glucose in the blood, resulting in a persistent increase in blood glucose. Blood glucose levels are regulated by glucose uptake and endogenous glucose. According to the literature, IR promotes gluconeogenesis pathways and is regulated by a series of transcription factors, which are considered an important part of glucose metabolism in the liver [23]. G6Pase and PEPCK are the two rate limiting enzymes in regulating blood glucose homeostasis during gluconeogenesis. PEPCK could catalyze the reaction between phosphoenolpyruvate and oxaloacetate. At the end of gluconeogenesis, G6Pase catalyzes glucose 6-phosphate to produce free glucose. Upregulated expression of PEPCK and G6Pase promotes gluconeogenesis. Thus, G6Pase and PEPCK expression is much higher in the liver of patients with T2DM than in healthy individuals [24]. We used two proteins, PEPCK and G6Pase, as entry points to verify whether EPS103 could inhibit gluconeogenesis by inhibiting the expression of gluconeogenesis-related enzymes, thus regulating blood glucose metabolism. As shown in Figure 5D–F, compared to the NC group, the expression of both PEPCK and G6Pase was significantly upregulated in the MC group (*p* < 0.05). The protein expression levels of PEPCK and G6Pase were significantly inhibited by EPS103 (*p* < 0.05). The effects were greater in the HD group than in the LD group; however, the treatment effects of different concentrations of EPS103 were not significantly different. In parallel, we also verified the expression of PGC-1*α* after intragastric treatment with EPS103 and metformin (Figure 5G); expression was also significantly downregulated, the decline was more pronounced in the HD group than in the LD and PC group, and there was also no difference in significance. The above data indicate that EPS103 can downregulate the T2DM mouse liver G6Pase, PEPCK and PGC-1*α* protein expression, and inhibit gluconeogenesis, thereby achieving a hypoglycemic effect.

### 3.7. The Effect of EPS103 on the IRS-1/PI3K/Akt Pathway

Insulin regulates the entire glycolipid metabolism process through the IRS-1/PI3K/Akt signaling pathway. After insulin binds to the insulin receptor, the insulin receptor bottom, IRS-1, is activated and binds to the downstream PI3K, which can further phosphorylate threonine/Akt. Activated Akt acts on the downstream signaling pathway to affect glycolipid metabolism [25]. The PI3K/Akt pathway plays a key role in insulin signaling. It has been shown to regulate various physiological processes associated with T2DM, such as stimulating glucose uptake in adipose tissue, muscle, and liver, in addition to promoting insulin signaling [26]. Studies have shown that Akt is an important signaling molecule in the insulin signaling pathway. It is said to be the center of the insulin signaling pathway, and phosphorylated Akt can also activate glycogen synthase GSK-3*β* [27,28]. IR would inhibit the insulin mediated tyrosine phosphorylation activity of IRS-1, which would further inhibit the PI3K/Akt signaling pathway and reduce the body’s use of glucose [29]. Meanwhile, the downstream protein, Akt, is phosphorylated by PI3K (p85), which promotes the transfer of GLUT4 to the cell surface through phosphorylation, thereby increasing the body’s uptake of glucose [30]. On this basis, GSK-3*β* is inhibited by Akt phosphorylation, downregulating glycogen synthase phosphorylation levels, increasing glycogen synthase activity, and then inducing glycogen synthesis.

The Tyr632 phosphorylation content of IRS-1 (0.7 ± 0.2) was significantly inhibited in the MC group (*p* < 0.05, Figure 5H,I). After administration with metformin and EPS103, the Tyr632 phosphorylation content of IRS-1 was significantly restored compared with the MC group (*p* < 0.05). Additionally, compared with the NC group, both the Tyr458 phosphorylation content of PI3K (0.65 ± 0.08) and the Ser473 phosphorylation content of Akt (0.62 ± 0.05) in the MC group were significantly inhibited (*p* < 0.05, Figure 5H,J). In the EPS103 and metformin treatment groups, the Tyr458 phosphorylation content of PI3K and the Ser473 phosphorylation content of Akt improved to different extents. In contrast, the recovery of PI3K’s Tyr458 phosphorylation after EPS103 treatment was significantly greater at different concentrations than after metformin treatment. The expression of Tyr458 content of PI3K (1.5 ± 0.4) in the HD group was significantly higher than other groups (*p* < 0.05). Meanwhile, In treatment groups Ser473 phosphorylation of Akt recovered to a degree close to that of the NC group (0.85 ± 0.09) (Figure 5H,K). From this, we concluded that EPS103 activates the IRS-1/PI3K/Akt signaling pathway in T2DM mice, which is inhibited by IR. Consistent with our study, Huang et al. (2013) found that EPS isolated from kimchi can activate the PI3K/Akt pathway to regulate important markers in glycometabolic processes [29].

### 3.8. EPS103 Alleviates Brain Nerve Damage of T2DM Mice

**Figure 6:**
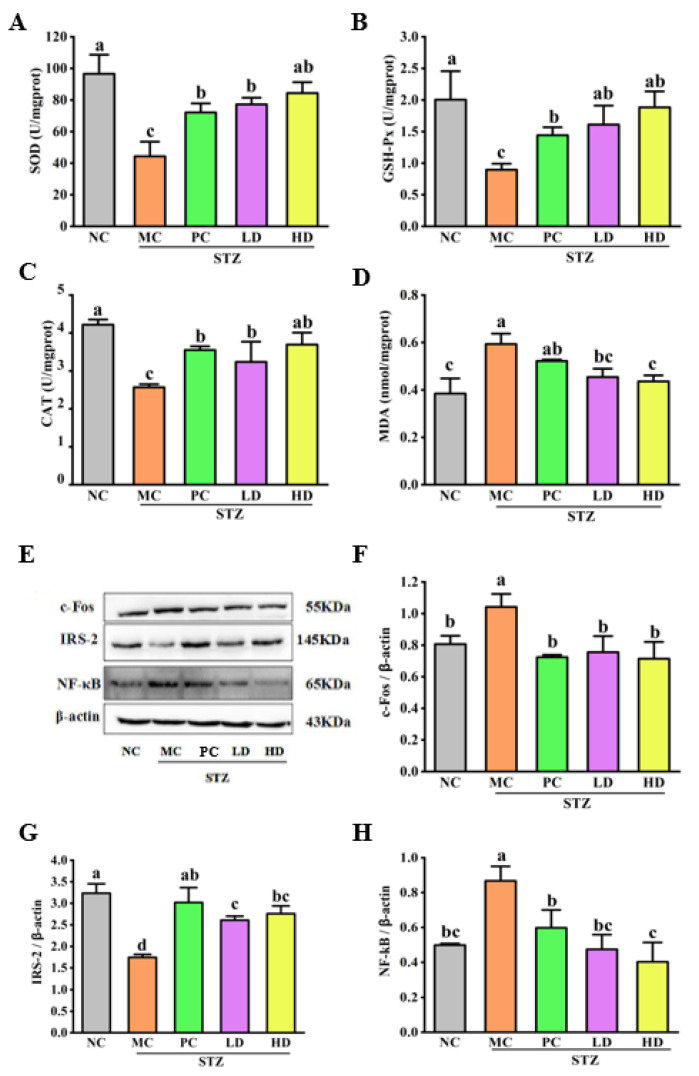
Effect of EPS103 on brain nerve damage in the T2DM mice induced by STZ and HFD. Activities of (**A**) SOD, (**B**) GSH-Px and (**C**) CAT, as well as content of (**D**) MDA in the hippocampus. (**E**) Bands of c-Fos, IRS-2 and NF-κB. Protein expression levels of (**F**) c-Fos, (**G**) IRS-2, and (**H**) NF-κB. *β*-actin was utilized as loading control. Data are presented as the mean ± SD (n = 3). Different letters indicate statistically significant differences between two groups (*p* < 0.05).

Oxidative stress is an important factor contributing to the functional decline of pancreatic islet *β* cells, and oxidative stress leads to the overproduction of reactive oxygen species (ROS) and reactive nitrogen species, in which reactive oxygen species can directly damage islet *β* cells [31]. Studies have shown that increased ROS is undoubtedly a crucial factor in the progression of diabetes and its complications [32]. In addition, numerous studies have shown that oxidative stress increases the production of free radicals in fat and muscle cells, decreases antioxidant substances, and increases the accumulation of oxidation products, such as MDA [33]. It has been proven that the increase in antioxidants such as SOD and GSH-Px can eliminate reactive oxygen species and further improve oxidative stress [34]. Simulating mice using STZ results in autophosphorylation of insulin receptors and increased activity of intrinsic tyrosine kinase, phosphotyrosine phosphatase, which in turn leads to inhibition of insulin signaling, disruption of glucose and energy metabolism in the brain, and induction of impaired consciousness. Furthermore, IR caused by STZ not only affects glucose metabolism in the hippocampus and cortex but also leads to Alzheimer’s disease (AD)-like pathological features, such as oxidative stress and synaptic dysfunction caused by the activation of microglia [35]. To understand whether T2DM would further lead to impaired brain hippocampal tissue function through oxidative stress, we performed an assay to determine antioxidant levels in hippocampal tissue of T2DM mice. As shown in Figure 6A–C, after HFD and STZ-induced T2DM mice, T2DM in the hippocampus of mice showed significantly lower SOD activity, lower GSH-PX activity, and higher MDA levels than NC group (*p* < 0.05). After treatment for 4 weeks, treatment groups (PC, LD and HD) resulted in significant enhancements in SOD, CAT, and GSH-Px activities relative to the MC group, differences among the treatment groups were not significant. Additionally, the level of MDA was significantly reduced (*p* < 0.05) compared with the levels found in the MC group. These findings indicate no significant differences between the EPS103 treatment groups (LD and HD) and MC group (*p* < 0.05, Figure 6D). It has been shown that polysaccharides from astragalus can regulate the balance between oxidizing lipids and antioxidant enzymes by altering the amount of MDA in brain tissue, while increasing SOD activity, and further alleviating neuronal damage and dysfunction in the brain. These results indicate that EPS103 can improve oxidative stress injury in the hippocampus of T2DM mice.

Cognitive and motor functions are regulated and maintained by the insulin signaling pathway in the central nervous system. Insulin receptors are abundantly expressed in various cell types in the brain, including neurons and oligodendrocytes [36]. T2DM and AD are impairments of insulin signaling pathways associated with low brain metabolism and cognitive dysfunction. IRS-2 is found in cells of all tissue types throughout the body, but mainly in the liver and pancreas. IRS-2 is not only abundantly expressed in the liver and pancreatic islet *β* cells, but is also a major docking protein in the islet signaling pathway in the CNS. It has been reported that the expression of IRS-2 in the brain is mainly localized in the hippocampus and cortex. The location and distribution of these insulin receptors, which are not only important for the organism’s stress response, blood glucose, etc., are consistent with the earliest sites of damage to vulnerable neurons in AD. IRS-2 is one of the key molecules in the insulin signaling pathway, and its abnormal expression can lead to blocked or attenuated insulin signaling pathways with IR appearance. Mouse MC groups showed significantly lower IRS-2 expression than NC mice (*p* < 0.05, Figure 6E,G). In contrast to the MC group, treatment with metformin and EPS103 significantly restored IRS-2 expression in the hippocampus of T2DM mice. These results indicate that EPS103 have potential repair effect on the insulin signaling in the hippocampus of T2DM mice.

Based on previous findings, it is generally accepted that c-Fos is an immediate early gene (IEG). It can be used as a marker of brain cell functionality; there is a close relationship between c-Fos expression and hippocampal structural alterations. Glutamate (Glu) is an important excitatory neurotransmitter within the central nervous system and the main excitatory neurotransmitter in the hippocampus. Glu can also promote c-Fos expression. Data from studies have shown that emergency injuries produce long-term increases in Glu levels in the hippocampus, which are significantly higher than the basal values. At the same time, excessive Glu release, together with activation of its receptors, further triggers neuronal necrosis. c-Fos gene expression is a hallmark of Glu receptor activation [37]. Neuronal activation caused by nociceptive stimuli can be detected by the expression of c-Fos. Measurement of c-Fos levels in T2DM mice’s hippocampal tissues allowed us to determine whether EPS103 could alleviate cerebral neuronal damage caused by IR. After the completion of T2DM modeling, the c-Fos content in the hippocampus of the mice in the MC group was significantly higher than that in the NC group (*p* < 0.05, Figure 6E,F), indicating that IR-induced neurons receive noxious stimuli and promote neuronal apoptosis in the mouse brain. Regarding the treatment groups (PC, LD and HD groups), the content of c-Fos in each group was significantly reduced (*p* < 0.05), and the therapeutic effect of each group allowed c-Fos to return to near-normal levels. These results indicate that EPS103 could alleviate neuronal apoptosis in the hippocampus of T2DM mice.

NF-κB plays an important regulator in transcription. It not only plays an important role in the body’s inflammatory and immune responses. In most published studies, but also effectively regulate cell apoptosis and stress response. In general, NF-κB is present in the cytoplasmic matrix in an inactive state and is associated with its protein inhibitor, IKB. When a cell is activated, NF-κB translocates into the nucleus, inducing the transcription of related genes. Hyperactivation is closely associated with many human diseases, such as rheumatoid arthritis and inflammatory changes in the heart and brain. Many studies have shown that inflammation plays a role in the pathogenesis and progression of diabetes as well as the decline of cognitive function. It has been observed in numerous studies that diabetes induced hippocampal neuron expression is significantly increased [38]. Therefore, drugs that inhibit the NF-κB signal transduction pathway may become an effective method for the treatment of chronic inflammation. Simultaneously, inhibiting the increase in NF-κB expression may be an important entry point in the treatment of cognitive impairment induced by diabetes. In this study, we investigated NF-κB expression in the hippocampal tissues of T2DM mice. The level of NF-κB expression in the MC group (0.86 ± 0.08) was significantly higher than the NC group (0.49 ± 0.01) (*p* < 0.05, Figure 6E,H), indicating excessive activation of NF-κB in the brains of T2DM mice. After treatment with EPS103, NF-κB expression level significantly decrease compared with the MC group (*p* < 0.05) and was close to the NC group. These results indicate that EPS103 can be functional factors because of their antioxidant and hypoglycemic properties. To sum up, oral administration of EPS103 can improve the oxidative stress injury and inhibit the inflammatory response, restore the expression of insulin receptor substrate, and effectively alleviate the brain nerve injury induced by HFD and STZ in T2DM mice.

## 4. Conclusions

The hypoglycemic effect of the EPS from L. plantarum JLAU103 (EPS103) on STZ and HFD-induced T2DM mice were evaluated. EPS103 can effectively relieve T2DM through ameliorating IR, reducing blood glucose concentration, improving glucose tolerance, protecting pancreas and liver, and repairing dyslipidemia. Moreover, EPS103 can promote hepatic glycogenesis and glucose uptake, and inhibit gluconeogenesis. The potential mechanisms are partially associated with the activation of the IRS-1/PI3K/AKT signal pathways. In addition, EPS103 can protect against brain nerve damage by improving oxidative stress injury, restoring the expression of IRS-2, alleviating neuronal apoptosis and inhibiting inflammation in the hippocampus of T2DM mice. These findings suggest that EPS103 has potential applications in the prevention and alleviation of T2DM. However, the structure–function relationship of EPS103 on hyperglycemia and hyperlipidemia needs further investigation.

## Figures and Tables

**Table 1:** Effect of EPS103 on fasting blood glucose levels of T2DM mice.

Groups	Fasting Blood Glucose Level (mmol/L)						
	Week 2	Week 6	Week 7	Week 8	Week 9	Week 10	Week 11
NC	4.6 ± 0.6 ^b^	5.3 ± 0.4 ^c^	5.4 ± 0.8 ^b^	5.7 ± 0.7 ^b^	5.4 ± 0.9 ^c^	5 ± 1 ^c^	5.8 ± 0.6 ^c^
MC	7.7 ± 0.6 ^a^	8.7 ± 0.5 ^b^	12.2 ± 0.7 ^a^	12.5 ± 0.9 ^a^	12.8 ± 0.6 ^a^	13.6 ± 1.1 ^a^	12 ± 1 ^a^
PC	8.1 ± 0.5 ^a^	11 ± 1 ^a^	13.1 ± 0.7 ^a^	12 ± 1 ^a^	11 ± 1 ^b^	8.8 ± 0.8 ^b^	8 ± 1 ^b^
LD	7 ± 1 ^a^	8.9 ± 0.8 ^ab^	12.9 ± 0.7 ^a^	12 ± 1 ^a^	11.1 ± 0.9 ^ab^	9.5 ± 0.7 ^b^	8 ± 1 ^b^
HD	8 ± 1 ^a^	10.2 ± 1.1 ^ab^	13.5 ± 0.9 ^a^	12.1 ± 0.9 ^a^	10.6 ± 0.4 ^b^	8.1 ± 0.8 ^b^	6.2 ± 0.7 ^bc^

Mean values in the same column with different letters had significance on statistics (*p <* 0.05).

**Table 2:** Effect of EPS103 on serum lipid concentrations of T2DM mice.

Groups	TG (mmol/L)	TC (mmol/L)	LDL-C (mmol/L)	HDL-C (mmol/L)
NC	0.7 ± 0.1 ^c^	3.7 ± 0.2 ^c^	0.6 ± 0.1 ^c^	2.84 ± 0.06 ^a^
MC	1.45 ± 0.07 ^a^	5.7 ± 0.1 ^a^	2.0 ± 0.2 ^a^	1.59 ± 0.09 ^d^
PC	0.76 ± 0.04 ^bc^	5.0 ± 0.2 ^b^	1.77 ± 0.05 ^a^	2.28 ± 0.09 ^bc^
LD	0.9 ± 0.1 ^b^	5.1 ± 0.1 ^b^	1.4 ± 0.1 ^b^	2.12 ± 0.07 ^c^
HD	0.81 ± 0.08 ^bc^	5.1 ± 0.1 ^b^	1.36 ± 0.08 ^b^	2.35 ± 0.05 ^b^

Mean values in the same column with different letters had significance on statistics (*p* < 0.05).

## Data Availability

The data presented in this study are available on request from the corresponding author.
